# Early and mid-term outcomes of off-pump versus on-pump coronary artery bypass surgery in patients with triple-vessel coronary artery disease: a randomized controlled trial

**DOI:** 10.1186/s13019-023-02258-6

**Published:** 2023-04-13

**Authors:** Seyed Mohammad Forouzannia, Seyed Khalil Forouzannia, Pourya Yarahmadi, Mohammad Alirezaei, Akbar Shafiee, Negin Yazdian Anari, Farzad Masoudkabir, Zahra Dehghani, Mina Pashang

**Affiliations:** 1 Tehran Heart Center, Cardiovascular Diseases Research Institute, Tehran University of Medical Sciences, North Kargar St, Tehran, 1411713138 Iran; 2 Cardiac Primary Prevention Research Center, Cardiovascular Diseases Research Institute, Tehran University of Medical Sciences, Tehran, Iran; 3 Faculty of Medicine, Shahid Sadoughi University of Medical Sciences, Yazd, Iran

**Keywords:** Coronary artery bypass grafting, Coronary artery bypass, off-pump, Cardiopulmonary bypass, Major adverse cardiac events, Mortality

## Abstract

**Background and aim of the study:**

Several studies have compared early and late outcomes of on-pump coronary artery bypass grafting (CABG) and off-pump CABG. However, there is still an ongoing debate on this matter, especially in patients with triple-vessel coronary artery disease (3VD).

**Methods:**

We randomly assigned 274 consecutive patients with 3VD to two equal groups to undergo on-pump CABG or off-pump CABG. The primary outcome was major adverse cardiac and cerebrovascular events (MACCE), including all-cause mortality, acute coronary syndrome, stroke or transient ischemic attack, and the need for repeat revascularization. The secondary outcomes were postoperative infection, ventilation time, ICU admission duration, hospital stay length, and renal failure after surgery.

**Results:**

The median follow-up duration was 31.2 months (range 24.6–35.2 months). The mean age of patients was 61.4 ± 9.3 years (range: 38–86), and 207 (78.7%) were men. There were 15 (11.2%) and 9 (7.0%) MACCE occurrences in on-pump and off-pump groups, respectively (*P *value = 0.23). MACCE components including all-cause death, non-fatal MI, CVA, and revascularization did not significantly differ between on-pump and off-pump groups. We observed no difference in the occurrence of MACCE between off-pump and on-pump groups in multivariable regression analysis (HR = 0.57; 95% CI 0.24–1.32; *P *value = 0.192). There were no statistical differences in postoperative outcomes between the off-pump and on-pump CABG groups.

**Conclusions:**

Off-pump CABG is an equal option to on-pump CABG for 3VD patients with similar rates of MACCE and postoperative complications incidence when surgery is performed in the same setting by an expert surgeon in both methods. (IRCT20190120042428N1).

## Introduction

Coronary artery bypass grafting (CABG) remains the most frequently performed procedure in adult cardiac surgery [1]. CABG is traditionally performed by utilizing cardiopulmonary bypass (on-pump CABG). Off-pump CABG was first introduced in the mid-1980s to reduce postoperative complications caused by cardiopulmonary bypass (CPB) and cross-clamping of the aorta during on-pump CABG [2, 3]. Since then, several randomized clinical trials [4–13] and observational studies [14, 15] have reported comparable early and/or late outcomes of on-pump and off-pump CABG. However, none of these two methods was found to be superior to the other, and their results are still debating the postoperative complications and major adverse cardiac and cerebrovascular events (MACCE) through various years of follow-up. This debate is especially more profound in patients with multiple coronary artery diseases [16–19]. Nonetheless, considerable diversities in the study design, main outcomes, and confounding adjustments in the literature cause severe heterogeneity and controversy among previous studies.

In this study, we aimed to shed more light on the priority of on-pump or off-pump CABG in three-vessel disease patients by investigating the impact of off-pump versus on-pump on early and late clinical outcomes.

## Methods

### Study design and population

The current study was a single-center randomized, controlled trial. symptomatic patients diagnosed with triple-vessel coronary artery disease (CAD) by angiography and were scheduled for isolated CABG surgery at Tehran Heart Center between April 2018 and April 2020 were included in the study. Patients with single-vessel CAD and/or other concomitant cardiac surgery, such as valve replacement, aorta reconstruction, or other additional cardiovascular disease necessitating concomitant surgery, were excluded. All medical records were retrieved from the Cardiac Surgery Database of the Tehran Heart Center. The study protocol was approved by Tehran Heart Center ethical board (IR.TUMS.THC.REC.1399.005) and was registered at the Iranian clinical trial registry (IRCT20190120042428N1). All patients signed written informed consent upon enrollment. This study was designed and performed under the declaration of Helsinki and its updates.

### Enrolment and randomization

Eligible patients were randomly assigned to two equal groups (off-pump CABG or on-pump CABG) by a blocked randomization scheme with a block size of four. Since the patient care team could not be blinded to the patient treatment group, blindness was considered for patients, follow-up coordinators, and data analysts.

### Surgical technique

All surgeries were performed by an expert and high-volume surgeon (S.K.F) with experience in more than 10,000 off-pump and 5,000 on-pump surgeries during the past 25 years. General anesthesia was induced and maintained with a fast-track cardiac anesthesia method. Patients were positioned, prepped, and draped in a standard fashion. Median sternotomy was used as the surgical access in all cases. Left internal mammary arteries (LIMA) were harvested in all cases, and saphenous veins were used as other conduits. In both off-pump and on-pump groups, cardiac displacement was achieved by placing two moisturized gauze pads (10 × 10 cm) between the pericardium and the left ventricle. This maneuver elevated and rotated the left ventricle toward the midsternal incision area, bringing the LAD into view. In the off-pump group, The chosen device for coronary artery stabilization was the Medtronic Octopus (Medtronic, Minneapolis, Minn). The target vessel was then opened, and an intracoronary shunt (Medtronic, Minneapolis, Minn) was introduced to prevent blood loss and maintain distal perfusion during the performance of anastomosis. The operative field was visualized using the carbon dioxide surgical blower system. Surgical revascularization was mainly started from LIMA to the left anterior descending artery (LAD) grafting. Following this, the right coronary system was approached, and the circumflex territory was finally revascularized. In patients with left main CAD, LAD, and circumflex arteries were always grafted regardless of the degree of stenosis. All other vessels with significant lesions (> 70%) were identified preoperatively in the angiogram and selected as a target for revascularization. All proximal anastomoses were performed using the side-biting aortic clamp and 6–0 polypropylene sutures. Distal anastomoses (LIMA-LAD and SVGs) were performed using 8–0 and 7–0 polypropylene suture, respectively. Sorin Stockert’s S3 Heart–Lung Machine was used for the conventional CPB for the on-pump group. The standard CPB technique was employed with ascending aortic cannulation and venous drainage via a 2-stage venous cannula within the right atrium with complete clamping of the aorta with cardioplegia arrest.

### Study outcomes

The primary outcomes of this study were the incidence of major adverse cerebro-cardiovascular events (MACCE), including all-cause mortality, non-fatal MI, stroke or transient ischemic attack, and the need for repeat revascularization (percutaneous coronary intervention or redo-CABG). The secondary outcomes were postoperative wound infection, ventilation time, duration of ICU admission, length of hospital stay, and renal failure after surgery.

### Follow-up

Patients were evaluated for the occurrence of study outcomes in one, three, and six months (by telephone) intervals and then annually after surgery. The surgeon performed face-to-face visits, and our center’s general practitioners did phone follow-ups. The first event was considered for survival analysis in cases with more than one event. All the baseline and follow-up information of the patients were recorded in the Tehran Heart Center cardiac surgery registry.

### Statistical analysis

All analyses were conducted according to the intention-to-treat principle. Continuous data were expressed as mean ± SD, and categorical data were expressed as percentages. Before further analysis, we checked the normality of data in the two groups using the Kolmogorov–Smirnov test, and the skewness and kurtosis indices were analyzed. Independent student’s t-test or Kruskal–Wallis test (for continuous variables) and Chi-square or Fisher exact tests (for categorical variables) were used for a comparative analysis of baseline characteristics and postoperative outcomes. To investigate the independent risk factors of MACCE, Variables with significant differences (*p* value < 0.10) between on-pump and off-pump groups were entered as confounding variables in multivariable Cox regression analysis. Kaplan–Meier using log-rank test were utilized to describe the time to the first occurrence of MACCE and all-cause mortality between off-pump and on-pump groups. Findings were reported as hazard ratio (HR) and 95% confidence interval (CI). All *P *values are 2-sided, and *P *values < 0.05 were considered significant. All statistical analyses were performed using STATA version 17.0 (College Station, TX: StataCorp LLC, USA).

## Results

### Characteristics

**Fig. 1:**
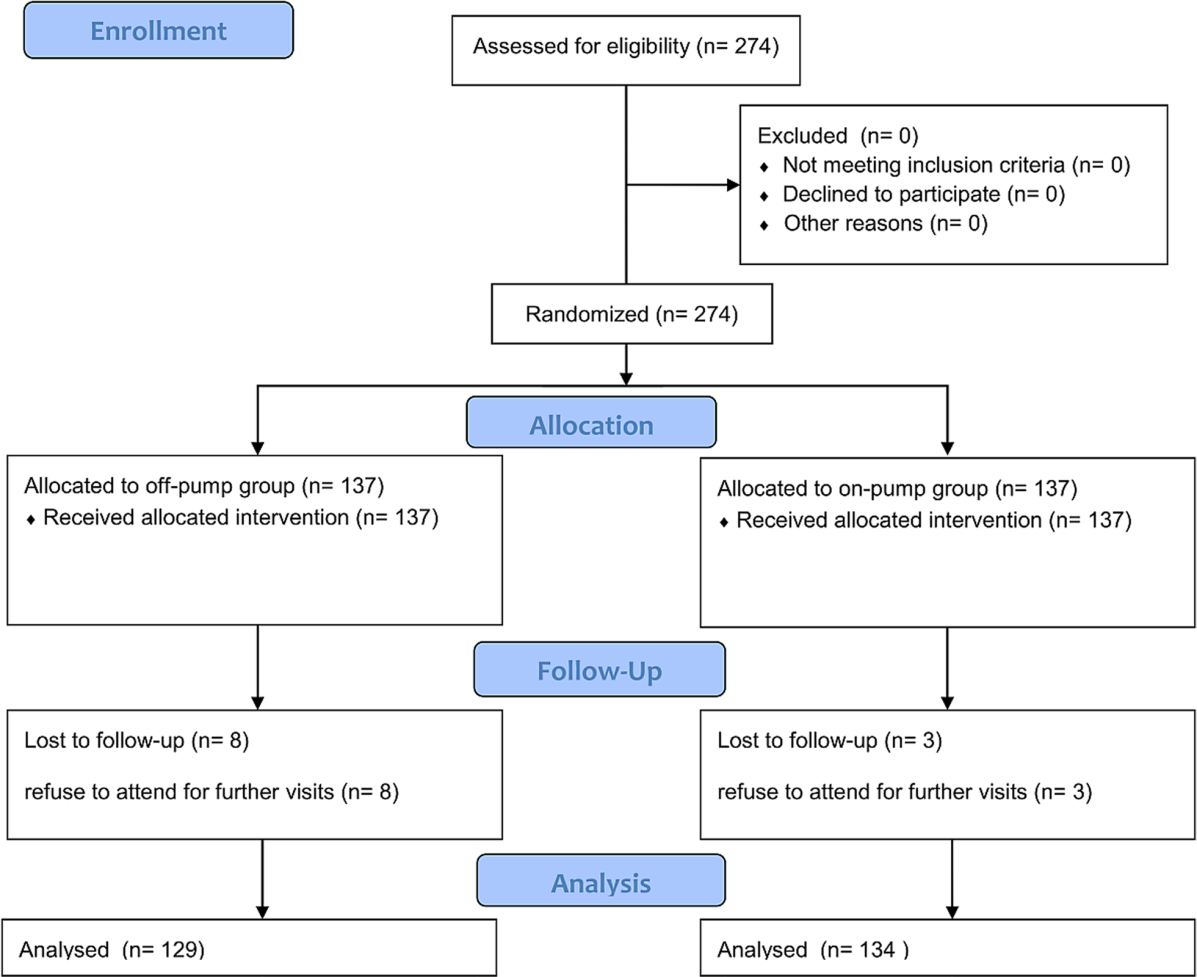
CONSORT flow diagram of the current study

Two hundred seventy-four patients were enrolled in our study. No one died due to in-hospital mortality, but 11 patients (4.1%) were lost to follow-up. No off-pump patient was converted to the on-pump method. Therefore, 263 patients were included in our analysis (134 off-pump and 129 on-pump) (Fig. 1). The median follow-up duration was 31.2 months (range of 24.6–35.2 months). The mean age of patients was 61.4 ± 9.3 years (range, 38 to 86), and 207 (78.7%) were men. There was no statistically significant difference in the baseline characteristics of on-pump and off-pump groups except for the left main coronary artery disease (*P *value = 0.002) and EF under 50% before CABG (*P *value = 0.033) (Table 1).

**Table 1:** Baseline demographic and clinical characteristics of the study population

Characteristics*#	Total (n = 263)	On-pump (n = 134)	Off-pump (n = 129)	*P* value†
Age, year	61.4 ± 9.4	62.3 ± 9.9	60.6 ± 8.8	0.135
Male	207 (78.7)	109 (81)	98 (76)	0.287
BMI, mg/m^2^	27.6 ± 8	27.9 ± 10.2	27.2 ± 4.6	0.442
Smoking				0.702
Current	52 (19.8)	28 (20.9)	24 (18.6)	
Former	19 (7.2)	11 (8.2)	8 (6.2)	
Never	192 (73)	95 (71)	97 (75.2)	
Opium				0.850
Current	23 (8.8)	11 (8.2)	12 (9.4)	
Former	7 (2.7)	3 (2.2)	4 (3.1)	
Never	232 (88.5)	120 (89.5)	112 (87.5)	
LVEF	44 ± 9.3	42.2 ± 10.3	45.8 ± 7.9	0.002
LVEF < 50% prior to surgery	157 (59.7)	88 (65.7)	69 (53.5)	0.044
Hypertension	150 (57)	76 (56.7)	74 (57.4)	0.915
Previous MI	68 (25.9)	32 (23.9)	36 (27.9)	0.456
Peripheral vascular disease	3 (1.3)	2 (1.7)	1 (0.9)	> 0.99
COPD	14 (5.4)	5 (3.8)	9 (7)	0.247
Diabetes mellitus	124 (47.5)	67 (50)	57 (44.9)	0.408
History of CVA	13 (4.9)	7 (5.2)	6 (4.6)	0.830
Left main disease	44 (16.7)	32 (23.9)	12 (9.3)	0.002
Dyslipidemia	115 (43.9)	56 (42.1)	59 (45.7)	0.554
Previous renal failure	15 (5.7)	8 (6)	7 (5.4)	0.859
Number of grafts	3.5 ± 0.6	3.5 ± 0.6	3.5 ± 0.6	0.901

*BMI* Body mass index, *CABG* Coronary artery bypass grafting, *COPD* Chronic obstructive pulmonary disease, *CVA* Cerebrovascular accident, *LVEF* Left ventricular ejection fraction, *MI* myocardial infarction

*Data were present as n (%) or mean ± SD

^#^Baseline characteristics had no or < 3% missing observations unless indicated. The percentages are calculated based on available data and may not add to 100%

^†^*P* value < 0.05 was considered statistically significant

### Major adverse cardiac events

There were 15 (11.2%) and 9 (7.0%) MACCE in on-pump and off-pump groups, respectively (*P *value = 0.23). MACCE components including all-cause mortality, non-fatal MI, CVA, and need for repeat revascularization did not significantly differ between on-pump and off-pump groups (Table 2). 13 (4.9%) cases of all-cause mortality were observed during follow-up. Seven (5.2%) patients in the on-pump group and six (4.6%) patients in the off-pump group died (*P *value = 0.830).

**Table 2:** Comparing the frequency of primary and secondary outcomes between on- and off-pump CABG groups

Outcome^*^	Total (n = 263)	On-pump (n = 134)	Off-pump (n = 129)	*P* value^#^
*Primary outcomes*				
Total MACCE, n (%)	24 (9.1)	15 (11.2)	9 (7.0)	0.235
All-cause death, n (%)	13 (4.9)	7 (5.2)	6 (4.6)	0.83
CVA, n (%)	3 (1.1)	2 (1.5)	1 (0.8)	> 0.999
MI, n (%)	7 (2.7)	5 (3.7)	2 (1.5)	0.447
Revascularization, n (%)	1 (0.4)	1 (0.7)	0 (0.0)	> 0.999
*Secondary outcomes*				
Wound infection, n (%)	7 (2.7)	5 (3.8)	2 (1.5)	0.447
Renal failure after CABG, n (%)	5 (1.9)	3 (2.2)	2 (1.5)	> 0.99
Ventilation, hours	11.5 [7.5]	12 [7]	11 [7]	0.527
ICU, hours	34.7 ± 30.1	32.6 ± 25.2	36.8 ± 34.4	0.256
Hospitalization, day	15.8 ± 7.5	16.1 ± 8.5	15.4 ± 6.5	0.467
Surgery to Discharge, day	6.9 ± 3.3	7.1 ± 3.7	6.8 ± 3.0	0.424

*CABG* Coronary Artery Bypass Grafting, *MACCE* Major Adverse Cardiac and Cerebrovascular Events, *CVA* Cerebrovascular Accident, *MI* Myocardial Infarction, *ICU* Intensive Care Unit

*Data were present as n (%) or mean ± SD or median [interquartile range]

^#^*P* < 0.05 was considered statistically significant

### Secondary outcomes

There were no statistical differences in postoperative outcomes between the off-pump and on-pump CABG groups (Table 2). Seven (2.7%) patients experienced wound infection (2.2% in on-pump and 1.5% in the off-pump group; *p* = 0.44) and renal failure was observed in five (1.5%) patients (2.2% in on-pump and 1.5% in the off-pump group; *p* > 0.999). In addition, ventilation time (*P *value = 0.72), duration of ICU admission (*P *value = 0.256), length of hospital stay (*P *value = 0.467), and surgery to discharge time (*P *value = 0.424) did not significantly differ between the two groups (Table 2).

### Multivariable analysis

**Fig. 2:**
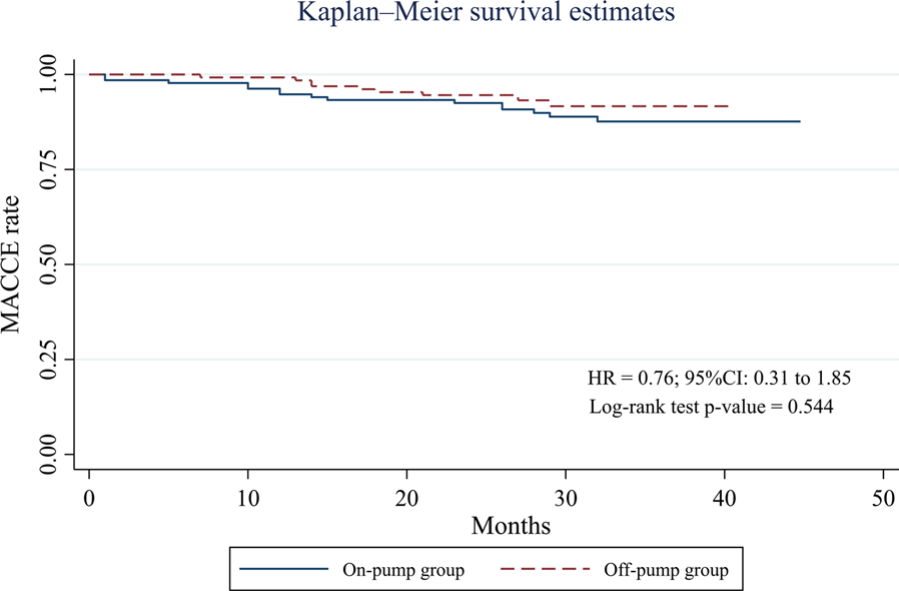
Kaplan–Meier for major adverse cardiac and cerebrovascular events (MACCE) rate comparing patients undergoing on- versus off-pump coronary artery bypass graft surgery

**Fig. 3:**
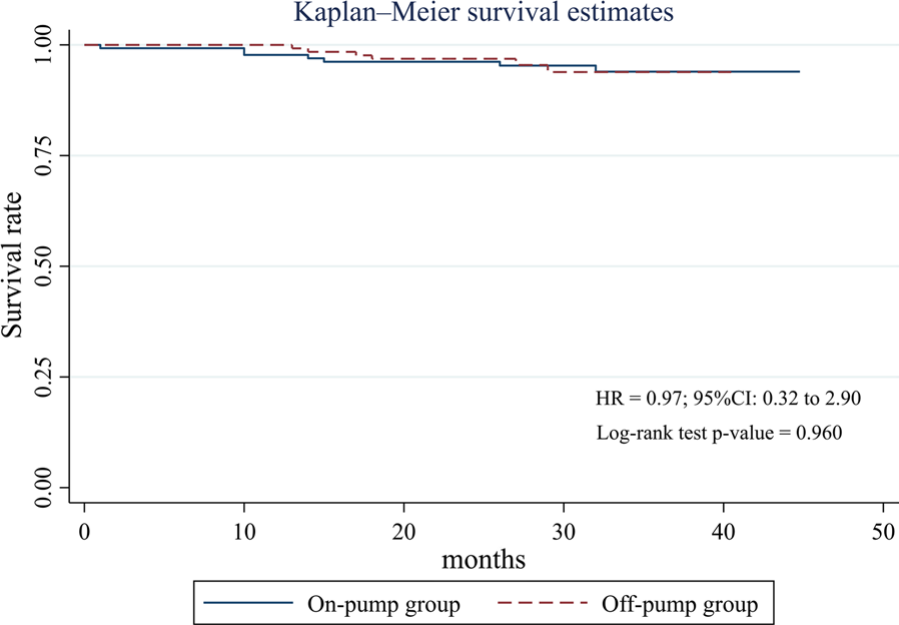
Kaplan–Meier for all-cause mortality rate comparing patients undergoing on- versus off-pump coronary artery bypass graft surgery

The frequency of left main CAD and EF under 50% before CABG significantly differed between the groups. Therefore, We determined the independent contribution of on-pump and off-pump CABG surgery on the incidence of MACCE by multivariable Cox regression. This analysis showed no difference in the occurrence of MACCE between off-pump and on-pump groups (HR = 0.57; 95% CI 0.24–1.32; *P *value = 0.192) (Table 3). Kaplan–Meier curves for all-cause death and the composite MACCE depicted no significant difference in the event-free survival for those undergoing an off-pump versus an on-pump procedure (*P *value = 0.960 for all-cause death and *P *value = 0.544 for the composite MACCE; Figs. 2 and 3).

**Table 3:** Predictors of major adverse cardiac and cerebrovascular events

Variable	Hazard ratio	95% confidence interval	*P *value*
Off-pump CABG	0.57	0.24–1.32	0.192
EF < 50% before CABG	0.48	0.21–1.08	0.078
Left main disease history	0.38	0.08–1.61	0.188

*CABG* Coronary artery bypass grafting, *EF* Ejection fraction

*P < 0.05 was considered statistically significant

## Discussion

This study compared off-pump versus on-pump postoperative and long-term MACCE outcomes in 263 three-vessel coronary artery disease patients who underwent CABG surgery. At a median follow-up of 31.2 months, the groups had no significant difference in postoperative outcomes. In addition, we found no significant difference in rates of MACCE incidence between the groups at the end of the follow-up.

Several studies have reported early and late outcomes between off-pump and on-pump during sequential follow-up intervals. However, their results are controversial in some aspects. Among large-scale trials, the CORONARY trial which is only one performed subgroup analysis for 3VD patients, reported no difference between off-pump and on-pump groups in MACCE outcomes after 30-days, 1-year, and 5-year follow-up in three distinct studies [5–7]. In addition, the MASS III trial with 5-year and 10-year follow-ups also reported no difference between groups in both follow-up intervals [12, 13]. On the contrary, the ROOBY trial showed a higher rate of MACCE at 1-year and 5-year with off-pump than on-pump CABG [9–11]. In addition, Benedetto et al. reported that off-pump surgery is associated with a significantly increased risk of 3-year all-cause death among patients with the left main disease (hazard ratio: 1.94; 95% confidence interval: 1.10 to 3.41; *P *value = 0.02) [20].

There were no differences in postoperative outcomes between the off-pump and on-pump CABG groups in the current study. In contrary with our results, the MASS III trial reported a longer length of ICU stay, time to extubation, and hospital stay in off-pump patients [13]. On the other hand, the CORONARY trial reported that off-pump surgery was associated with shorter operations and shorter duration of ventilator support and reduced rates of postoperative complications such as reoperation for perioperative bleeding, respiratory complications, and acute kidney injury [6]. We presume there will be no difference in the rate of postoperative complications between off-pump and on-pump CABG when the surgery is performed by an expert surgeon and heart team in both methods.

There are important differences between these trials, which these controversies may stem from. Different sample sizes, diverse clinical settings and eligibility criteria, primary outcomes, surgeon experience, and duration of follow-up can be mentioned as the most frequent reasons. Variations in eligibility criteria of previous studies, such as enrollment of high-risk patients [21], number of grafts [22], age [23, 24], history of left main CAD [25], and ventricular dysfunction [26, 27] could be another source of heterogeneity in the result of these studies.

There is a trend of fewer completed grafts than originally planned in the off-pump group compared with the on-pump group in previous trials. CORONARY, ROOBY, and MASS III trials reported fewer grafts and/or a higher rate of incomplete revascularization (as assessed by the surgeon at the time of surgery) among the off-pump group [6, 11, 13]. In the current study number of grafts was equal between the off-pump and on-pump groups (3.5 ± 0.6 vs. 3.5 ± 0.6; respectively) and the same number of grafts as proposed before surgery was performed for all patients.

In the present study, all surgeries were done by a high-volume surgeon who is an expert in both methods. Squires et al. reported a correlation between surgeon experience and long-term outcomes and reduced mortality of off-pump among high-volume surgeons [28]. In addition, surgeon experience is an essential factor in operative mortality. Benedetto et al. reported that the lack of experience in the off-pump technique would increase the conversion rate (off-pump to on-pump) and operative risk [29]. Nevertheless, there is no consensus among recent trials about the definition of a high-volume surgeon or center [30].

Follow-up duration plays a vital role in the detection of MACCE. Reported follow-up durations varied among previous studies [31]. Current study endpoints (with a median follow-up of 31.2 months) are comparable to high-quality trials with at least 3-years follow-up duration, suggesting no differences in composite MACE between off-pump and on-pump groups[5, 13, 23]. MASS III reported no difference between the groups in composite MACE incidence at a 10-year follow-up, consistent with the results of this trial at a 5-year follow-up [12]. However, there is a controversy among studies with longer follow-ups. A meta-analysis of 16 observational studies with more than ten years of follow-up reported that off-pump CABG is associated with a higher mortality rate than the on-pump group (HR = 1.07, 95% CI 1.03–1.12, *P *value = 0.0008) [32]. In addition, there are few studies with longer follow-up duration [19], and this issue demands further research.

Our trial has some limitations, according to the small sample size of the current study compared to large-scale trials, results need to be confirmed by subgroup analysis of 3VD patients in previous trials or further studies focused on this population. The present report does not include data on long-term morbidity and mortality. However, further follow-up and angiographic control of graft patency are planned, and the results will be published in the future.

## Conclusion

In conclusion, off-pump CABG is an equal option to on-pump CABG with a similar rate of all-cause death, non-fatal stroke, non-fatal myocardial infarction, or recurrent revascularization in 3VD patients when surgery is performed in the same setting by the same high-volume surgeon in both methods. In addition, no significant difference was observed in postoperative infection, renal failure after CABG, need for ventilation, ICU admission, and hospitalization duration.

## Data Availability

All data included in this study are available from the corresponding author upon E-mail request.

## Abbreviations

BMI: Body mass index
CABG: Coronary artery bypass grafting
COPD: Chronic obstructive pulmonary disease
CVA: Cerebrovascular accident
LVEF: Left ventricular ejection fraction
MI: Myocardial infarction
MACCE: Major adverse cardiac and cerebrovascular events
ICU: Intensive care unit

## References

[1] Bowdish ME, D’Agostino RS, Thourani VH, Desai N, Shahian DM, Fernandez FG (2020). The society of thoracic surgeons adult cardiac surgery database: 2020 update on outcomes and research. Ann Thorac Surg.

[2] Kowalewski M, Pawliszak W, Malvindi PG, Bokszanski MP, Perlinski D, Raffa GM (2016). Off-pump coronary artery bypass grafting improves short-term outcomes in high-risk patients compared with on-pump coronary artery bypass grafting: meta-analysis. J Thorac Cardiovasc Surg.

[3] Deppe A-C, Arbash W, Kuhn EW, Slottosch I, Scherner M, Liakopoulos OJ (2016). Current evidence of coronary artery bypass grafting off-pump versus on-pump: a systematic review with meta-analysis of over 16 900 patients investigated in randomized controlled trials. Eur J Cardiothorac Surg.

[4] Puskas JD, Williams WH, Duke PG, Staples JR, Glas KE, Marshall JJ (2003). Off-pump coronary artery bypass grafting provides complete revascularization with reduced myocardial injury, transfusion requirements, and length of stay: a prospective randomized comparison of two hundred unselected patients undergoing off-pump versus conventional coronary artery bypass grafting. J Thorac Cardiovasc Surg.

[5] Lamy A, Devereaux PJ, Prabhakaran D, Taggart DP, Hu S, Straka Z (2016). Five-year outcomes after off-pump or on-pump coronary-artery bypass grafting. N Engl J Med.

[6] Lamy A, Devereaux PJ, Prabhakaran D, Taggart DP, Hu S, Paolasso E (2012). Off-pump or on-pump coronary-artery bypass grafting at 30 days. N Engl J Med.

[7] Lamy A, Devereaux PJ, Prabhakaran D, Taggart DP, Hu S, Paolasso E (2013). Effects of off-pump and on-pump coronary-artery bypass grafting at 1 year. N Engl J Med.

[8] Houlind K, Kjeldsen BJ, Madsen SN, Rasmussen BS, Holme SJ, Nielsen PH (2012). On-pump versus off-pump coronary artery bypass surgery in elderly patients: results from the Danish on-pump versus off-pump randomization study. Circulation.

[9] Quin JA, Wagner TH, Hattler B, Carr BM, Collins J, Almassi GH (2022). Ten-year outcomes of off-pump vs on-pump coronary artery bypass grafting in the department of veterans affairs: a randomized clinical trial. JAMA Surg.

[10] Shroyer AL, Hattler B, Wagner TH, Collins JF, Baltz JH, Quin JA (2017). Five-year outcomes after on-pump and off-pump coronary-artery bypass. N Engl J Med.

[11] Shroyer AL, Grover FL, Hattler B, Collins JF, McDonald GO, Kozora E (2009). On-pump versus off-pump coronary-artery bypass surgery. N Engl J Med.

[12] Hueb W, Rezende PC, Gersh BJ, Soares PR, Favarato D, Lima EG (2019). Ten-year follow-up of off-pump and on-pump multivessel coronary artery bypass grafting: MASS III. Angiology.

[13] Hueb W, Lopes NH, Pereira AC, Hueb AC, Soares PR, Favarato D (2010). Five-year follow-up of a randomized comparison between off-pump and on-pump stable multivessel coronary artery bypass grafting. The MASS III trial. Circulation.

[14] Kirmani BH, Holmes MV, Muir AD (2016). Long-term survival and freedom from reintervention after off-pump coronary artery bypass grafting: a propensity-matched study. Circulation.

[15] Hannan EL, Wu C, Smith CR, Higgins RS, Carlson RE, Culliford AT (2007). Off-pump versus on-pump coronary artery bypass graft surgery: differences in short-term outcomes and in long-term mortality and need for subsequent revascularization. Circulation.

[16] Benedetto U, Caputo M, Mariscalco G, Gaudino M, Chivasso P, Bryan A (2017). Impact of multiple arterial grafts in off-pump and on-pump coronary artery bypass surgery. J Thorac Cardiovasc Surg.

[17] Yu L, Gu T, Shi E, Wang C, Fang Q, Yu Y (2014). Off-pump versus on-pump coronary artery bypass surgery in patients with triple-vessel disease and enlarged ventricles. Ann Saudi Med.

[18] Kim HJ, Chung JE, Jung JS, Kim IS, Son HS (2018). Current status of off-pump coronary artery bypass grafting in patients with multiple coronary artery disease compared with on-pump coronary artery bypass grafting: the Korean National Cohort study. Thorac Cardiovasc Surg.

[19] Raja SG, Garg S, Soni MK, Rochon M, Marczin N, Bhudia SK (2020). On-pump and off-pump coronary artery bypass grafting for patients needing at least two grafts: comparative outcomes at 20 years. Eur J Cardiothorac Surg.

[20] Benedetto U, Puskas J, Kappetein AP, Brown WM, Horkay F, Boonstra PW (2019). Off-pump versus on-pump bypass surgery for left main coronary artery disease. J Am Coll Cardiol.

[21] Lemma MG, Coscioni E, Tritto FP, Centofanti P, Fondacone C, Salica A (2012). On-pump versus off-pump coronary artery bypass surgery in high-risk patients: operative results of a prospective randomized trial (on-off study). J Thorac Cardiovasc Surg.

[22] Kim HJ, Chung JE, Jung JS, Kim IS, Son HS (2018). Current status of off-pump coronary artery bypass grafting in patients with multiple coronary artery disease compared with on-pump coronary artery bypass grafting: the Korean national cohort study. Thorac Cardiovasc Surg.

[23] Diegeler A, Börgermann J, Kappert U, Hilker M, Doenst T, Böning A (2019). Five-year outcome after off-pump or on-pump coronary artery bypass grafting in elderly patients. Circulation.

[24] Sun L, Zhou M, Ji Y, Wang X, Wang X (2022). Off-pump versus on-pump coronary artery bypass grafting for octogenarians: a meta-analysis involving 146 372 patients. Clin Cardiol.

[25] Modolo R, Chichareon P, Kogame N, Dressler O, Crowley A, Ben-Yehuda O (2019). Contemporary outcomes following coronary artery bypass graft surgery for left main disease. J Am Coll Cardiol.

[26] Ueki C, Miyata H, Motomura N, Sakaguchi G, Akimoto T, Takamoto S (2016). Off-pump versus on-pump coronary artery bypass grafting in patients with left ventricular dysfunction. J Thorac Cardiovasc Surg.

[27] Wang W, Wang Y, Piao H, Li B, Wang T, Li D (2019). Early and medium outcomes of on-pump beating-heart versus off-pump CABG in patients with moderate left ventricular dysfunction. Braz J Cardiovasc Surg.

[28] Squiers JJ, Schaffer JM, Banwait JK, Ryan WH, Mack MJ, DiMaio JM (2021). Long-term survival after on-pump and off-pump coronary artery bypass grafting. Ann Thorac Surg.

[29] Benedetto U, Lau C, Caputo M, Kim L, Feldman DN, Ohmes LB (2018). Comparison of outcomes for off-pump versus on-pump coronary artery bypass grafting in low-volume and high-volume centers and by low-volume and high-volume surgeons. Am J Cardiol.

[30] Puskas JD, Gaudino M, Taggart DP (2019). Experience is crucial in off-pump coronary artery bypass grafting. Am Heart Assoc.

[31] Gaudino M, Benedetto U, Bakaeen F, Rahouma M, Tam DY, Abouarab A (2018). Off-versus on-pump coronary surgery and the effect of follow-up length and surgeons’ experience: a meta-analysis. J Am Heart Assoc.

[32] Takagi H, Ando T, Mitta S (2017). Meta-analysis comparing ≥10-year mortality of off-pump versus on-pump coronary artery bypass grafting. Am J Cardiol.

